# From desert flora to cancer therapy: systematic exploration of multi-pathway mechanisms using network pharmacology and molecular modeling approaches

**DOI:** 10.3389/fphar.2024.1345415

**Published:** 2024-04-11

**Authors:** Adel Alblihy

**Affiliations:** ^1^ Medical Center, King Fahad Security College (KFSC), Riyadh, Saudi Arabia; ^2^ Department of Criminal Justice and Forensic Sciences, King Fahad Security Collage, Riyadh, Saudi Arabia

**Keywords:** Saudi Arabian flora, ovarian cancer, active constituents, multi-target drug discovery, molecular docking, MD simulation

## Abstract

Ovarian cancer, often labeled a “silent killer,” remains one of the most compelling and challenging areas of cancer research. In 2019 alone, a staggering 222,240 new cases of ovarian cancer were reported, with nearly 14,170 lives tragically lost to this relentless disease. The absence of effective diagnostic methods, increased resistance to chemotherapy, and the heterogeneous nature of ovarian cancer collectively contribute to the unfavorable prognosis observed in the majority of cases. Thus, there is a pressing need to explore therapeutic interventions that offer superior efficacy and safety, thereby enhancing the survival prospects for ovarian cancer patients. Recognizing this potential, our research synergizes bioinformatics with a network pharmacology approach to investigate the underlying molecular interactions of Saudi Arabian flora (*Onopordum heteracanthum*, *Acacia ehrenbergiana*, *Osteospermum vaillantii*, *Cyperus rotundus*, *Carissa carandas*, *Carissa spinarum*, and *Camellia sinensis*) in ovarian cancer treatment. At first, phytoconstituents of indigenous flora and their associated gene targets, particularly those pertinent to ovarian cancer, were obtained from open-access databases. Later, the shared targets of plants and diseases were compared to identify common targets. A protein–protein interaction (PPI) network of predicted targets was then constructed for the identification of key genes having the highest degree of connectivity among networks. Following that, a compound–target protein–pathway network was constructed, which uncovered that, namely, hispidulin, stigmasterol, ascorbic acid, octopamine, cyperene, kaempferol, pungenin, citric acid, d-tartaric acid, beta-sitosterol, (−)-epicatechin gallate, and (+)-catechin demonstrably influence cell proliferation and growth by impacting the AKT1 and VEGFA proteins. Molecular docking, complemented by a 20-ns molecular dynamic (MD) simulation, was used, and the binding affinity of the compound was further validated. Molecular docking, complemented by a 20-ns MD simulation, confirmed the binding affinity of these compounds. Specifically, for AKT1, ascorbic acid showed a docking score of −11.1227 kcal/mol, interacting with residues Ser A:240, Leu A:239, Arg A:243, Arg C:2, and Glu A:341. For VEGFA, hispidulin exhibited a docking score of −17.3714 kcal/mol, interacting with Asn A:158, Val A:190, Gln B:160, Ser A:179, and Ser B:176. To sum up, both a theoretical and empirical framework were established by this study, directing more comprehensive research and laying out a roadmap for the potential utilization of active compounds in the formulation of anti-cancer treatments.

## 1 Introduction

Ovarian cancer is a prevalent malignancy among females worldwide and is ranked eighth in terms of incidence (Kamilova and Valijonova, 2023). Ovarian cancer ranks as the seventh leading cause of cancer-linked mortalities, exerting a profound impact on socioeconomic and community health at a global level (Torre et al., 2017). Among gynecological malignancies, ovarian cancer exhibits the highest mortality rate in developed countries across the world (Raab et al., 2020). Additionally, ovarian cancer accounted for approximately 3% of all cancers occurring in women. Currently, the lifetime ovarian cancer risk is approximately 1 in 72 (Yang et al., 2017; Lisio et al., 2019). Studies have reported that the pathogenesis of ovarian cancer is affected by various endocrine factors such as pituitary gonadotropins, namely, luteinizing hormone and follicle-stimulating hormone, in addition to progesterone, androgens, insulin-like growth factor 1, and estrogens (Choi et al., 2007; Li et al., 2021). Moreover, the receptor expression of progesterone, estrogen, and androgen receptors has been proposed as a possible determinant for predicting the prognosis and chemosensitivity of ovarian cancer patients toward platinum agents (Li et al., 2021). Despite recent advancements in medical science, individuals affected by ovarian cancer continue to experience high morbidity rates, and currently available treatment options do not provide satisfactory outcomes. Consequently, higher death rates and the limited availability of effective treatment strategies have garnered significant attention from researchers worldwide. Given the grave statistics, with an estimated 314,000 new cases of ovarian cancer and 207,000 deaths annually worldwide, ovarian cancer stands as the most lethal gynecological malignancy (Sideris et al., 2024). Alarmingly, in Australia, the incidence of ovarian cancer cases and deaths is projected to increase by 42% and 55%, respectively, by the year 2040, underscoring an urgent need for effective therapeutic strategies. Therefore, researchers are increasingly concentrating on pharmacological interventions that incorporate natural agents. These agents present a promising avenue for ovarian cancer treatment, offering the potential to overcome limitations associated with current therapies, such as drug resistance and adverse side effects. Phytochemicals, with their diverse bioactive compounds, offer a rich repository for the identification of novel anticancer molecules with minimal side effects compared to conventional chemotherapy agents. The integration of bioinformatics tools further enhances this process by enabling the rapid and precise analysis of vast biochemical datasets, facilitating the identification of potential lead compounds (Sharma et al., 2022; Swain et al., 2023). These computational methods allow for the prediction of compound–target interactions, bioactivity, and the pharmacokinetic properties of phytochemicals, streamlining the drug development process. Notably, bioinformatics applications in network pharmacology and molecular docking studies provide invaluable insights into the mechanistic pathways of cancer, helping pinpoint specific targets for therapeutic intervention (Noor et al., 2023). By leveraging the synergy between phytochemical potential and advanced bioinformatic techniques, researchers can significantly expedite the discovery and development of effective, targeted treatments for ovarian cancer, setting the stage for the establishment of novel lead molecules with optimized therapeutic profiles.

Although the Arabian Peninsula is known for its arid climate and limited biodiversity, the Kingdom of Saudi Arabia boasts a diverse range of flora, encompassing a variety of trees, herbs, and shrubs, including many edible and medicinal plants (Rahman et al., 2004). The extensive land area of Saudi Arabia comprises diverse geographical landscapes and climates, resulting in a wider range of plant distribution throughout the country (Aati et al., 2019). The indigenous uses and pharmacological activity of these plants in Saudi Arabia demonstrate the strong relationship between local remedies, diet, health, and traditional healing practices unique to their cultures. Previous studies provide shreds of evidence that indigenous plants in Saudi Arabia, including *Onopordum heteracanthum* (Gouda et al., 2014), *Acacia ehrenbergiana* (Makeen et al., 2020), *Osteospermum vaillantii* (Gouda et al., 2014), *Cyperus rotundus* (AlQathama et al., 2022), *Carissa carandas* (Alshehri, 2020), *Carissa spinarum* (AlQathama et al., 2022), and *Camellia sinensis* (Dou et al., 2021), have anti-cancer properties for ovarian cancer. Plants found in these regions have been used by local inhabitants for treating a wide range of ailments. However, the mechanism by which these plants exert their therapeutic effects remains poorly understood.

Recognizing the potential of active compounds, Hopkins (2007) introduced an innovative *in silico* approach known as “network pharmacology” that recognizes the potential of active compounds based on network perspectives. Network pharmacology has emerged as a valuable tool in the drug-designing process, aiding the resurgence of traditional knowledge (Noor et al., 2022a). This method serves as a standard for the preliminary identification of small molecules and the discovery of new treatment options, thereby enhancing our understanding of the disease pathophysiology (Batool et al., 2022; Noor et al., 2023). As a result, network pharmacology had a profound impact on the revival of herbal medicines, leading to a major transformation in the process of pharmaceutical discovery. In summary, comprehending the intricate landscape of network pharmacology is crucial to successfully identifying candidate drugs (Noor et al., 2022b).

By merging network pharmacology with bioinformatics, an in-depth examination of the therapeutic effects of local Saudi plants on ovarian cancer was explored. This analysis was further validated using docking methodologies to predict binding affinities among phytoconstituents and target proteins. Later, molecular dynamic (MD) simulations shed light on intricate conformational shifts and the potential interactions within molecular complexes. Notably, this research offered groundbreaking revelations about the therapeutic capacities of native flora in tackling ovarian cancer, serving as a beacon for subsequent explorations in the domain.

## 2 Materials and methods

### 2.1 Screening of the plant-derived compounds

Screening of plant-related small molecules is considered an initial step in understanding the multi-target effect of medicinal plants against diseases. In the current study, database and literature search were carried out for obtaining the main active constituents of plants. In the literature and databases, the search was confined to *“O. heteracanthum*,*” “A. ehrenbergiana*,*” “O. vaillantii*,*” “C. rotundus*,*” “C. carandas*,*” “C. spinarum*,*” and “C. sinensis*.*”* PubChem (https://pubchem.ncbi.nlm.nih.gov/) and Google Scholar were considered for the extensive literature survey, while the phytochemical databases, including KNApSAcK (http://www.knapsackfamily.com/KNApSAcK/) (Shinbo et al., 2006) and IMPPAT (https://cb.imsc.res.in/imppat/home) (Mohanraj et al., 2018), were searched for obtaining the active compounds of selected plants. After retrieval, the drug-like potential of active compounds was analyzed by predicting their oral bioavailability (OB) and drug-likeness (DL) values. In pharmacology, OB denotes the fraction of small molecules that successfully enter the systemic circulation after an oral dose, thereby enabling them to exert their desired pharmacological effects (Pathak and Raghuvanshi, 2015). A minimum OB of 30% is generally employed as a benchmark criterion for screening potential drug candidates since it indicates that a considerable portion of the oral usage of small molecules is capable of being absorbed and reaching systemic circulation. Conversely, small molecules with OB < 30% are likely to exhibit limited efficacy due to poor absorption. Thus, a higher OB value is positively correlated with drug effectiveness and is a key determinant of drug development and optimization. In a similar vein, DL analysis is employed to assess the potential of a molecule to serve as an oral drug by evaluating its bioavailability using qualitative measures (Jia et al., 2020). Following that, the DL and OB values of compounds were obtained using Molsoft (https://molsoft.com/mprop/) (James et al., 2015) and SwissADME software (https://www.swissadme.ch/) (Daina et al., 2017), and only those compounds having OB and DL values greater than 30% and 18% were considered the finalist compounds. Furthermore, the molecular weight and two-dimensional (2D) structure of predicted phytoconstituents were collected from PubChem (Kim et al., 2019) and Molinspiration (https://molinspiration.com/) (Molinspiration, 2011).

### 2.2 ADMET profiling

Although DL and OB are key elements in evaluating the drug-like potential of a compound, it is important to note that these parameters do not serve as the sole determinants of a compound’s effectiveness for further development. ADMET properties, which encompass absorption, distribution, metabolism, excretion, and toxicity, are equally important factors that must be evaluated to assess a compound’s safety and efficacy (Rehman et al., 2022). The evaluation of ADMET properties is conducted during the initial phases of drug development, which substantially plays an essential role in predicting the potential safety, effectiveness, and pharmacokinetics of a drug. To this end, the SwissADME server (https://www.swissadme.ch/) (Daina et al., 2017) and the ProTox II tool (http://tox.charite.de/protox_II) (Banerjee et al., 2018) were used to assess the ADMET properties of small biologically active molecules. Small molecules demonstrating excellent absorption, favorable solubility properties, and low toxicity were selected for further analysis.

### 2.3 Prediction of known targets of active compounds

To determine the integrative efficacy of the compounds derived from local plants in Saudi Arabia, we used two different platforms, namely, STITCH (http://stitch.embl.de/) (Kuhn et al., 2007) and SwissTargetPrediction (Gfeller et al., 2014) databases, with a species limitation of “*Homo sapiens*.” Furthermore, the SMILES representation of the chosen ingredients was used as input for the SwissTargetPrediction (https://www.swisstargetprediction.ch/) platform. Specifically, only proteins with a probability > 0.7 were considered putative targets of Saudi plants. For the STITCH database, targets exhibiting a combined score >0.7 were considered the significant targets of particular compounds.

### 2.4 Identification of disease-related targets through microarray data analysis

Microarray data analysis is a technique used for the identification of genes that are differentially expressed among different conditions (Noor et al., 2021; Sufyan et al., 2021). This involves comparing the gene expression levels of samples from disease and control groups and calculating statistical measures to determine which genes are significantly differentially expressed. In this study, gene expression data were acquired from the NCBI–GEO database (https://www.ncbi.nlm.nih.gov/) (Barrett et al., 2005). The search term “Ovarian Cancer” was used to retrieve four microarray datasets, namely, GSE54388, GSE69428, GSE36668, and GSE40595. The selection of datasets was based on their inclusion of both affected and normal ovaries; the dataset must have >3 samples. After data selection, the gene expression data were processed through the limma package of R (Ritchie et al., 2015) for the screening of differentially expressed genes (DEGs) as disease-specific targets for ovarian cancer.

### 2.5 Construction of the compound–target network

Upon identifying putative targets associated with both ovarian cancer and local plants, a Venn diagram was created to determine overlapping proteins. Subsequently, these identified genes were regarded as potential targets of indigenous Saudi Arabian plants, holding promise as potential biomarkers for intercepting the pathophysiological processes associated with ovarian cancer. Additionally, Cytoscape version 3.8 (https://cytoscape.org/) (Smoot et al., 2011) was used to construct an active ingredient–target network based on these overlapping genes. Within the compound–target network, the nodes indicated the small molecules along with their corresponding targets, while gray dotted lines depicted the possible interactions among these nodes. Finally, the NetworkAnalyzer plugin was used to evaluate the connectivity of targets exhibited by the chemical constituents among networks.

### 2.6 Gene Ontology and KEGG enrichment analyses

After identifying the common target, our study categorizes these targets based on their Gene Ontology (GO). GO covers three aspects of biology: cellular components (CCs), molecular functions (MFs), and biological processes (BPs). The BP term describes the protein’s role in various biological activities, and the CC term signifies the precise intracellular localization of a protein. Concurrently, the MF records the specific molecular tasks and interactions in which the protein or gene is involved. To achieve this, the current study used the DAVID database (https://david.ncifcrf.gov/tools.jsp) (Dennis et al., 2003) for GO and Kyoto Encyclopedia for Genes and Genomes (KEGG) pathway analyses. However, to filter-out those with significant relevance, a statistical cutoff of *p*-value<0.05 was implemented.

### 2.7 Construction and analysis of the protein–protein interaction network

The common targets were then analyzed using the STRING database (https://string-db.org/) (von Mering et al., 2003) to produce a protein–protein interaction (PPI) network. PPIs exhibit high specificity, adaptability, and versatility and are, therefore, of considerable significance. PPI network common genes were then analyzed using Cytoscape version 3.8 (https://cytoscape.org/) (Smoot et al., 2011) to identify hub genes. Hub genes exhibit a high number of interactions with other proteins and are considered essential elements of the network as they contribute to its integrity and stability. Furthermore, central or ‘hub’ genes hold significance in a range of disease-linked pathways and biological processes, suggesting their pivotal role in diverse cellular functions. In the current study, we examined the topology of the PPI network to identify ‘hub’ genes. Metrics such as degree, closeness, and betweenness centrality can facilitate this process. Specifically, we employed the degree methods available in cytoHubba to discern these hub genes.

### 2.8 Compound–target–pathway network construction

The effects of Saudi Arabian flora on ovarian cancer were investigated by constructing compound–target and target protein–pathway networks using Cytoscape version 3.8 (https://cytoscape.org/) (Smoot et al., 2011). By merging these networks, a final compound–target–pathway network was subsequently established. The networks comprise various nodes and edges, where nodes represent pathways, compounds, and targets relevant to the disease, while edges indicate the interactions among these nodes. The compound–target–pathway network offers valuable insights regarding the synergistic activity of phytoconstituents in ovarian cancer treatment.

### 2.9 Molecular docking analysis

Using advanced molecular docking methodologies, intricate interactions between small molecules and anticipated protein structures were analyzed. This rigorous analytical procedure illuminated potential pharmacological combinations, suggesting potential synergistic responses for precision therapeutics. Initially, the protein structures were obtained from the RCSB PDB database, which were then carefully prepared for docking. Missing atoms, residues, and side chains were addressed using the protein preparation tools within Chimera, ensuring a complete and accurate representation of the protein’s conformation. Water molecules and non-essential ions were removed, and hydrogen atoms were added to the protein structures to reflect physiological pH conditions. After this refinement, the proteins were subjected to an energy minimization process using the steepest descent and conjugate gradient methods, terminating at an energy convergence threshold of 0.01 kcal/mol to attain stable conformations. Subsequent docking studies were conducted using AutoDock Vina 1.1.2 implemented within the PyRx 0.8 interface (https://sourceforge.net/projects/pyrx/) (Dallakyan and Olson, 2015). The SMILES strings for the phytoconstituents were sourced from the PubChem database and converted to 3D structures using Open Babel, which is integrated into the PyRx platform. A comprehensive energy minimization was configured for a total of 2000 steps to ensure the reliability of the ligand conformations prior to the docking simulations. Later, the active site residues of the target proteins were uncovered with the assistance of the CASTp tool (http://sts.bioe.uic.edu/castp/index.html?201l) (Dundas et al., 2006). PyRx 0.8 was employed for target docking, which facilitated the calculation of binding affinities between the small molecules and target proteins. In our study, docking scores were used as indicators of binding affinity between the identified phytochemicals and target proteins. A docking score of less than −5.00 kcal/mol was considered to indicate strong binding affinity, while a score of less than −7.00 kcal/mol was interpreted as signifying very strong binding affinity. These thresholds were based on established computational chemistry conventions and were used to prioritize compounds for further analysis. The docked complexes were then visualized employing Discovery Studio (https://discover.3ds.com/discovery-studio-visualizer-download) (Biovia, 2017), PyMOL (https://pymol.org/2/) (Seeliger and de Groot, 2010), and ChimeraX (https://www.cgl.ucsf.edu/chimerax/) (Pettersen et al., 2021) programs for a better understanding of the binding interactions.

### 2.10 Molecular dynamic (MD) simulation

All-atom molecular dynamics (MD) simulation is a computational approach that precisely represents each atom and bond within a system, facilitating an in-depth analysis of molecular dynamics (Sahoo et al., 2020). This method calculates the movements of every atom in a system, which are determined by the interactions amongst them, represented by interatomic potentials (Sahoo et al., 2022). In this research, to execute the MD simulations of the ultimate complexes, we implemented the OPLS-AA/L force field using GROMACS 2018 software. The three-dimensional configurations of the protein served as the initial structural inputs for the simulations. To enhance their quality and suitability for the simulations, additional optimization was performed using Dock Prep (Pettersen et al., 2004). The parameterization of the active ingredients was executed using the SwissParam webserver (Zoete et al., 2011). Subsequently, MD simulations were conducted for a duration of 20 nanoseconds (ns), following a methodology similar to previous studies (Alamri, 2020). Various general MD simulation parameters were assessed for each complex, including root mean square deviation (RMSD), radius of gyration (Rog), and root mean square fluctuation (RMSF) (Needle et al., 2015). These parameters provide important information about the stability, conformational changes, and flexibility of the complexes during the simulation period.

## 3 Results

### 3.1 Screening of active compounds

The screening of active compounds represents a critical initial step in the identification of promising phytoconstituents with potential therapeutic efficacy against ovarian cancer. After searching and filtering, four compounds, namely, hispidulin, apigenin, luteolin, and beta-sitosterol were obtained from *O. heteracanthum*; two compounds, namely, stigmasterol and ascorbic acid were obtained from *A. ehrenbergiana*; five compounds namely, octopamine, 3,4-dihydroxybenzoic acid, cyperene, rotundine A, and rotundine B were obtained from *O. vaillantii*; six compounds namely, orientin, quercetin, kaempferol, pungenin, rotundine B, and cyperene were obtained from *C. rotundus*; nine compounds namely, carissic acid, alpha1-sitosterol, citric acid, d-tartaric acid, ascorbic acid, beta-ionone, anethole, ursolic acid, and beta-sitosterol were obtained from *C. carandas*; six compounds, namely, carissic acid, kaempferol, quercetin, digitoxigenin, ursolic acid, and beta-sitosterol were obtained from *C. spinarum*; and four compounds, namely, (−)-epicatechin gallate, (+)-catechin, quercetin, and kaempferol were obtained from *C. sinensis.* These 36 compounds were considered potential phytoconstituents of local Saudi plants as they fulfill the criteria of molecular weight <500 g/mol, DL ≥ 0.18, and OB ≥ 0.30 (Table 1).

**TABLE 1:** Pharmacological and molecular properties of phytochemicals.

Plant source	Phytochemical	Oral bioavailability> 0.30	Drug-likeness>0.18	Molecular weight (g/mol)	PubChem ID
*Onopordum heteracanthum*	Hispidulin	0.55	0.46	300.26	5281628
	Apigenin	0.55	0.39	270.24	5280443
	Luteolin	0.55	0.38	286.24	5280445
	Beta-sitosterol	0.55	0.78	414.7	222284
*Acacia ehrenbergiana*	Stigmasterol	0.55	0.62	412.7	5280794
	Ascorbic acid	0.56	0.74	176.12	54670067
*Osteospermum vaillantii*	Octopamine	0.55	0.54	153.18	4,581
	3,4-Dihydroxybenzoic acid	0.56	0.23	154.12	72
	Cyperene	0.55	0.18	204.35	12308843
	Rotundine A	0.55	0.37	231.33	10728239
	Rotundine B	0.55	0.75	233.35	21603505
*Cyperus rotundus*	Orientin	0.55	0.59	448.4	5281675
	Quercetin	0.55	0.52	302.23	5280343
	Kaempferol	0.55	0.5	286.24	5280863
	Pungenin	0.55	0.54	314.29	12314759
	Rotundine B	0.55	0.75	233.35	21603505
	Cyperene	0.55	0.18	204.35	12308843
*Carissa carandas*	Carissic acid	0.55	0.66	456.7	73242193
	Alpha1-sitosterol	0.55	0.47	426.7	9548595
	Citric acid	0.56	0.52	192.12	311
	d-Tartaric acid	0.56	0.59	150.09	439655
	Ascorbic acid	0.56	0.74	176.12	54670067
	Beta-ionone	0.55	0.33	192.3	638014
	Anethole	0.55	0.29	148.2	637563
	Ursolic acid	0.85	0.66	456.7	64945
	Beta-sitosterol	0.55	0.78	414.7	222284
*Carissa spinarum*	Carissic acid	0.85	0.66	456.7	73242193
	Kaempferol	0.55	0.5	286.24	5280863
	Quercetin	0.55	0.52	302.23	5280343
	Digitoxigenin	0.55	0.93	374.5	4369270
	Ursolic acid	0.85	0.66	456.7	64945
	Beta-sitosterol	0.55	0.78	414.7	222284
*Camellia sinensis*	(−)-Epicatechin gallate	0.55	0.93	442.4	107905
	(+)-Catechin	0.55	0.64	290.27	9,064
	Quercetin	0.55	0.52	302.23	5280343
	Kaempferol	0.55	0.5	286.24	5280863

### 3.2 ADMET profiling

The chosen compounds were subjected to ADME analysis, and 12 compounds, namely, hispidulin, stigmasterol, ascorbic acid, octopamine, cyperene, kaempferol, pungenin, citric acid, d-tartaric acid, beta-sitosterol, (−)-epicatechin gallate, and (+)-catechin, were identified as the main active constituents, with local good GI absorption and limited permeability of BBB, as presented in Table 2. Within the scope of ADMET analysis, hepatotoxicity refers to the capacity of compounds to inflict harm on the liver, potentially resulting in liver malfunction or complete failure. Notably, all selected compounds were found to have inactive hepatotoxicity in the current study. On the other hand, carcinogenicity characterizes the potential to induce cancer, while mutagenicity denotes its capacity to provoke DNA alterations that could lead to developmental anomalies, mutations in cancer, or many others. Compared to our results, the final active ingredients demonstrated themselves to be non-mutagenic and non-carcinogenic. In sum, these outcomes further support that the native plants of Saudi Arabia have drug-like potential that could be integral to preventing and treating diseases.

**TABLE 2:** ADMET profiling of active compounds.

Plant source	Active compound	GI absorption	BBB permeant	P-gp substrate	CYP1A2 inhibitor	CYP2C19 inhibitor	CYP2C9 inhibitor	CYP2D6 inhibitor	CYP3A4 inhibitor	Log *K*_*p*_ * *(skin permeation)	Hepatotoxicity	Carcinogenicity	Mutagenicity	Cytotoxicity
*Onopordum heteracanthum*	Hispidulin	High	✗	✗	✓	✗	✗	✓	✓	−6.01 cm/s	✗	✗	✗	✗
	Beta-sitosterol	Low	✗	✗	✗	✗	✗	✗	✗	−2.20 cm/s	✗	✗	✗	✗
*Acacia ehrenbergiana*	Stigmasterol	Low	✗	✗	✗	✗	✓	✗	✗	−2.74 cm/s	✗	✗	✗	✗
	Ascorbic acid	High	✗	✗	✗	✗	✗	✗	✗	−8.54 cm/s	✗	✗	✗	✗
*Osteospermum vaillantii*	Octopamine	High	✗	✗	✗	✗	✗	✗	✗	−7.87 cm/s	✗	✗	✗	✗
	Cyperene	Low	✗	✗	✗	✓	✓	✗	✗	−4.48 cm/s	✗	✗	✗	✗
*Cyperus rotundus*	Kaempferol	High	✗	✗	✓	✗	✗	✗	✗	−6.70 cm/s	✗	✗	✗	✗
	Pungenin	Low	✗	✗	✗	✗	✗	✗	✗	−9.40 cm/s	✗	✗	✗	✗
	Cyperene	Low	✗	✗	✗	✓	✓	✗	✗	−4.48 cm/s	✗	✗	✗	✗
*Carissa carandas*	Citric acid	Low	✗	✗	✗	✗	✗	✗	✗	−8.69 cm/s	✗	✗	✗	✗
	d-Tartaric acid	Low	✗	✗	✗	✗	✗	✗	✗	−8.55 cm/s	✗	✗	✗	✗
	Ascorbic acid	High	✗	✗	✗	✗	✗	✗	✗	−8.54 cm/s	✗	✗	✗	✗
	Beta-sitosterol	Low	✗	✗	✗	✗	✗	✗	✗	−2.20 cm/s	✗	✗	✗	✗
*Carissa spinarum*	Kaempferol	High	✗	✗	✓	✗	✗	✗	✗	−6.70 cm/s	✗	✗	✗	✗
	Beta-sitosterol	Low	✗	✗	✗	✗	✗	✗	✗	−2.20 cm/s	✗	✗	✗	✗
*Camellia sinensis*	(−)-Epicatechin gallate	Low	✗	✗	✗	✗	✗	✗	✗	−7.91 cm/s	✗	✗	✗	✗
	(+)-Catechin	High	✗	✓	✗	✗	✗	✗	✗	−7.82 cm/s	✗	✗	✗	✗
	Kaempferol	High	✗	✗	✓	✗	✗	✗	✗	−6.70 cm/s	✗	✗	✗	✗

### 3.3 Known therapeutic targets acting on ovarian cancer

**FIGURE 1:**
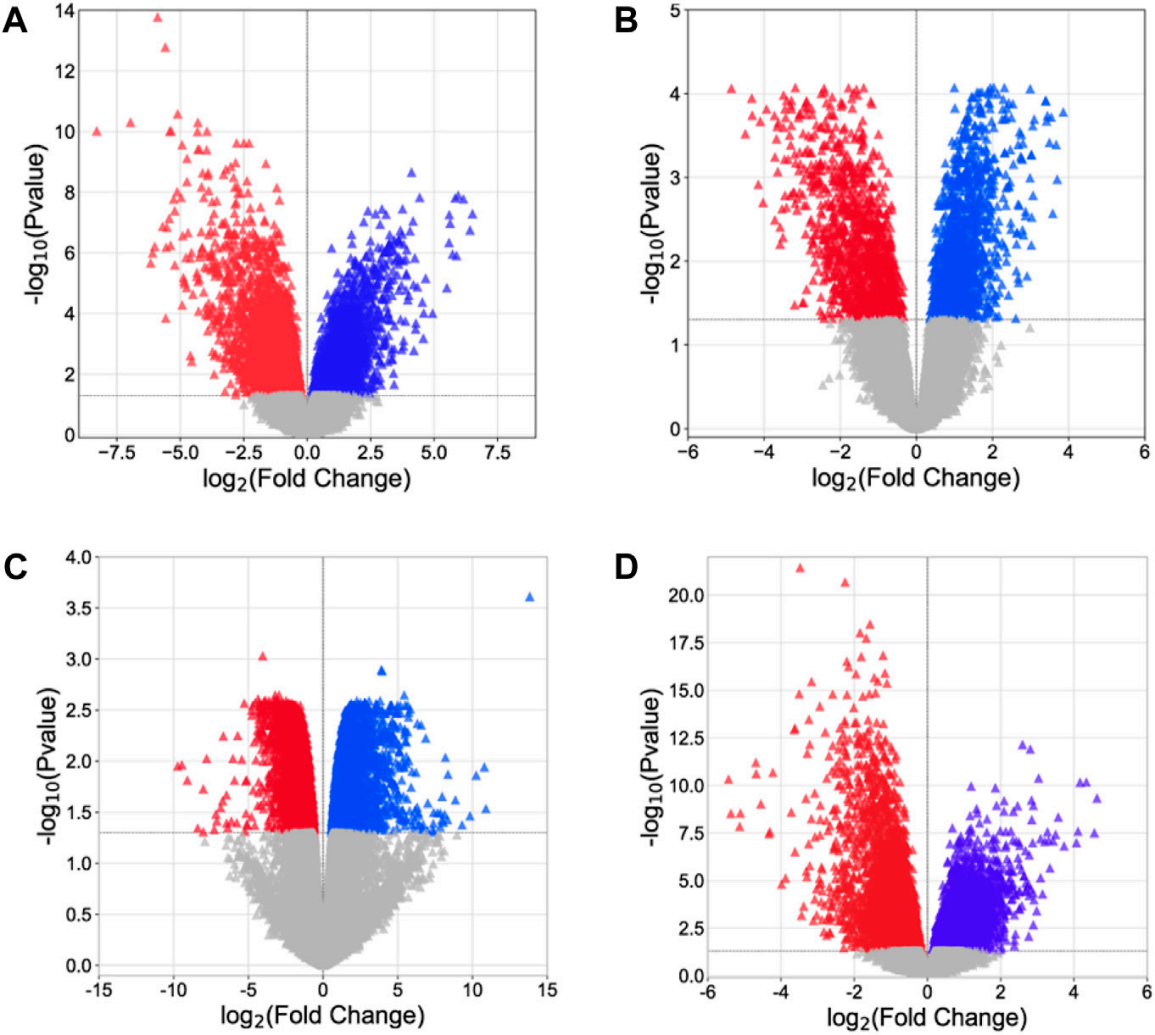
Volcano plot. **(A)** GSE54388, **(B)** GSE69428, **(C)** GSE36668, and **(D)** GSE40595. The red triangle represents downregulated genes, the blue triangle indicates the upregulated genes, and the gray triangle represents the non-significant genes.

Analysis of microarray data offers crucial insights into the molecular processes associated with disease progression and assists in the identification of possible targets for therapeutic intervention. Four gene expression datasets, namely, GSE54388, GSE69428, GSE36668, and GSE40595, were obtained from the NCBI–GEO database. Subsequent processing for the detection of DEGs was carried out using the limma package (Table 3, Supplementary Material S4: Supplementary Tables S1–S3). Within the limma package, the LogFC >1.0 and *p*-value <0.05 criteria were set for the screening of DEGs. From the GSE54388 dataset, a total of 4,687 DEGs were obtained (2,457 upregulated and 2,230 downregulated), 2,084 DEGs (1,020 upregulated and 1,064 downregulated) from GSE69428, 6,924 DEGs (3,147 downregulated and 3,777 upregulated) from GSE36668, and 2,840 DEGs (1,423 upregulated and 1,417 downregulated) from the GSE40595 dataset (Figure 1, Supplementary Material S5). The identified DEGs were subsequently used as therapeutic targets for ovarian cancer.

**TABLE 3:** Brief summary of GEO datasets used in the current study along with obtained DEGs.

GEO dataset	Total sample	Affected	Control	Upregulated gene	Downregulated gene	Total DEG
GSE54388	22	16	6	2,457	2,230	4,687
GSE69428	20	10	10	1,020	1,064	2084
GSE36668	8	4	4	3,147	3,777	6,924
GSE40595	77	63	14	1,423	1,417	2,840

### 3.4 Construction of the compound–target network

Following the prediction of disease-associated targets, a Venn tool was employed to uncover shared genes between plant-associated targets and disease-related targets. SwissTargetPrediction and STITCH databases yielded 469 potential targets of active compounds (Supplementary Material S1: Supplementary Table S1). Finally, 200 targets were found to be common among both plants and diseases (Supplementary Material S2: Supplementary Table S1). Compounds targeting these 200 genes were then imported into Cytoscape. This network suggests that these 200 identified targets could potentially work in synergy when these indigenous Saudi plants are used as an anticancer remedy.

### 3.5 GO and KEGG enrichment analyses

**FIGURE 2:**
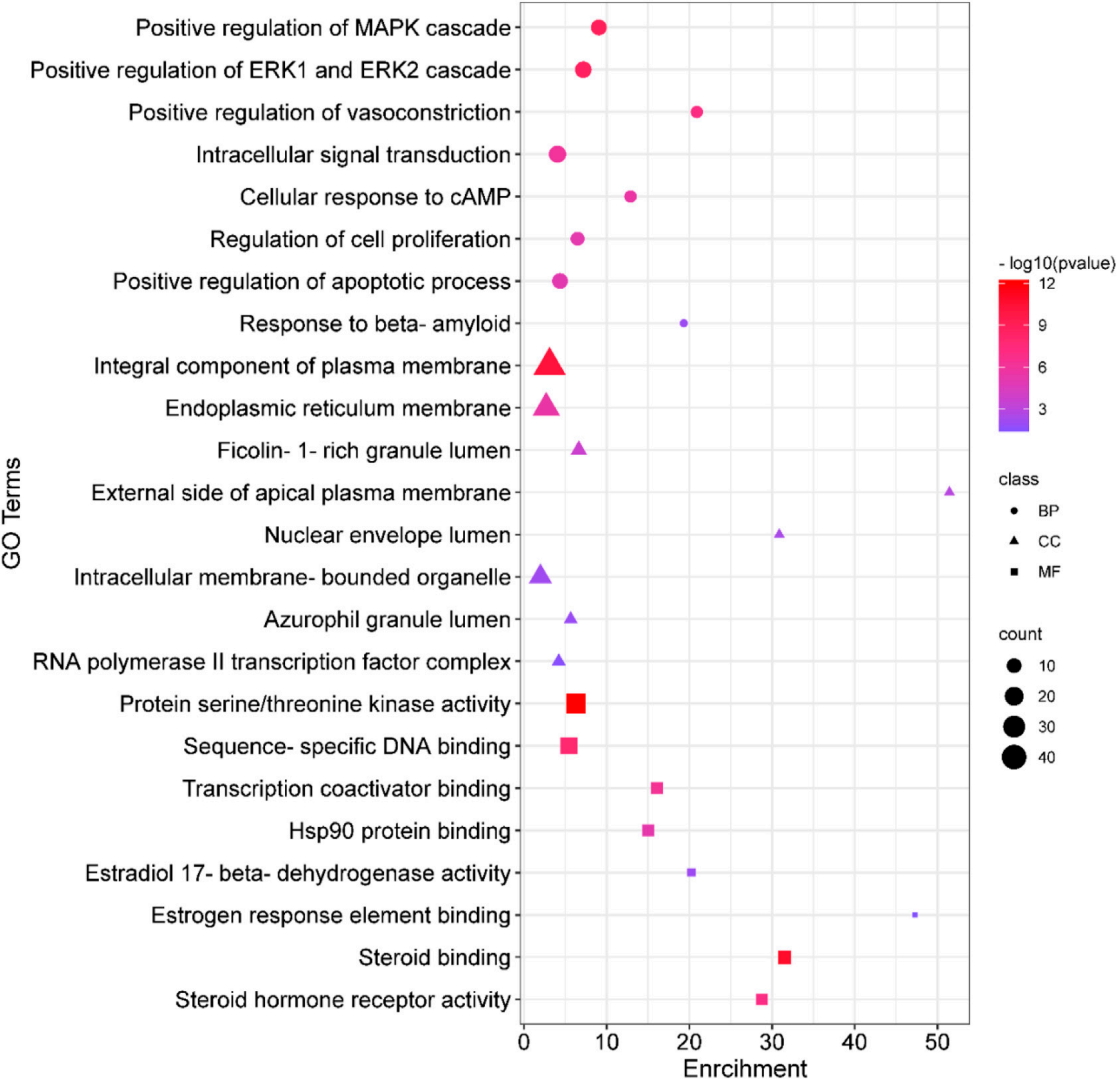
Gene Ontology (GO) enrichment analysis. In the plot, squares denote molecular functions, triangles depict cellular components, and circles signify biological processes. The size of each shape reflects the count, while the color indicates the *p*-value.

**FIGURE 3:**
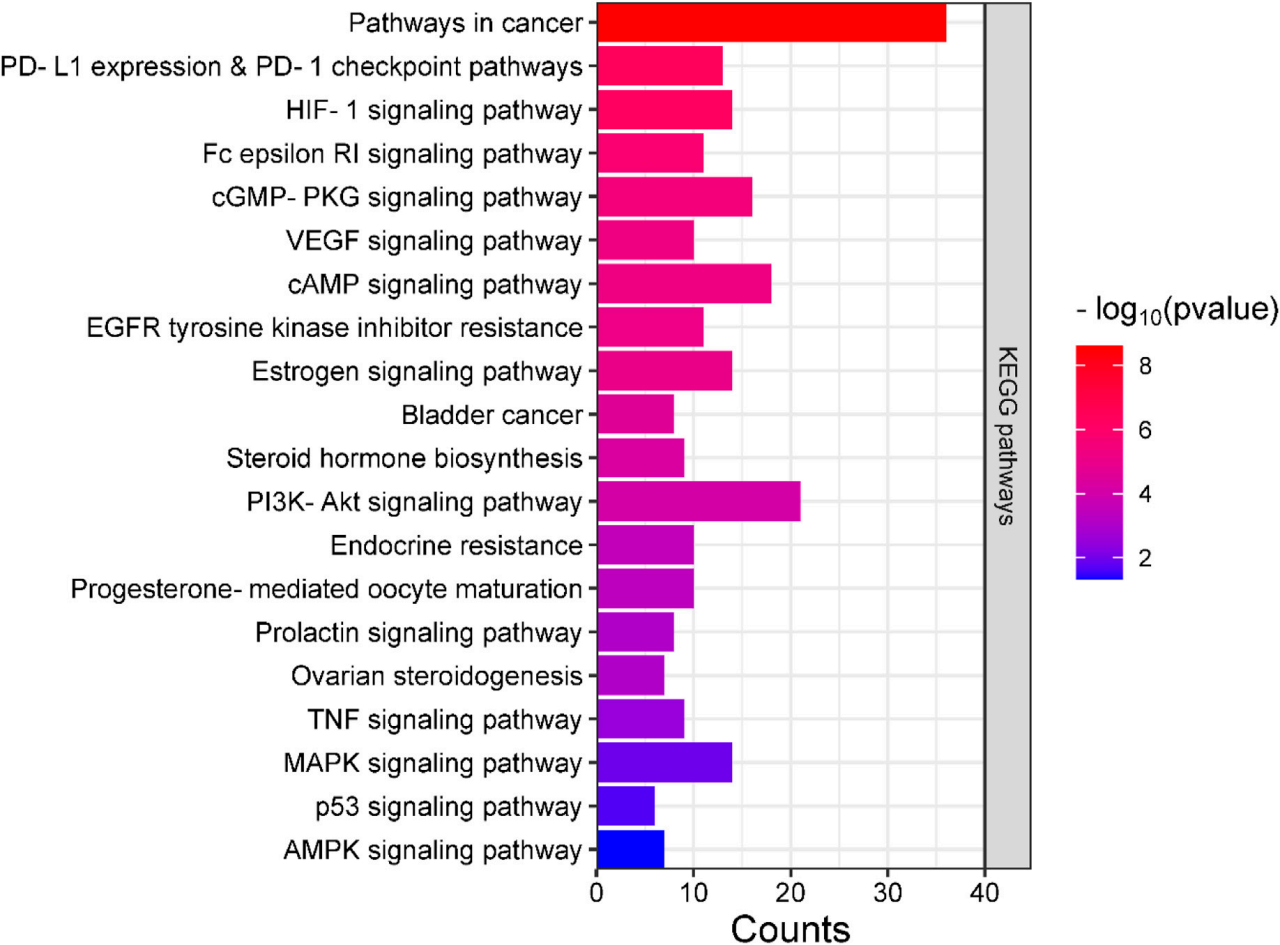
Bar plot illustrating the key KEGG pathways where the intersecting genes are predominantly concentrated.

Analyses for GO and pathway enrichment of common 200 target genes were carried out employing the DAVID tool to discern their biological characteristics (Supplementary Material S3: Supplementary Tables S1–S4). A total of 388 BP, 57 CC, and 109 MF terms met the *p*-value criterion of <0.05. Analysis of the top GO terms suggested that the shared genes were primarily associated with the positive regulation of the MAPK, ERK1, and ERK2 cascade, positive regulation of vasoconstriction, intracellular signal transduction, regulation of cell proliferation, positive regulation of the apoptotic process, Hsp90 protein binding, estradiol 17-beta-dehydrogenase activity, estrogen response element binding, steroid binding, and steroid hormone receptor activity (Figure 2). A total of 103 KEGG pathways were identified, indicating that the intersecting targets were predominantly involved in the mechanisms of growth hormone action, secretion, and synthesis, pathways in cancer, the estrogen signaling pathway, pI3K-Akt and ErbB signaling pathways, progesterone-mediated oocyte maturation, endometrial cancer, and many others (Figure 3).

### 3.6 Identification of hub genes

**FIGURE 4:**
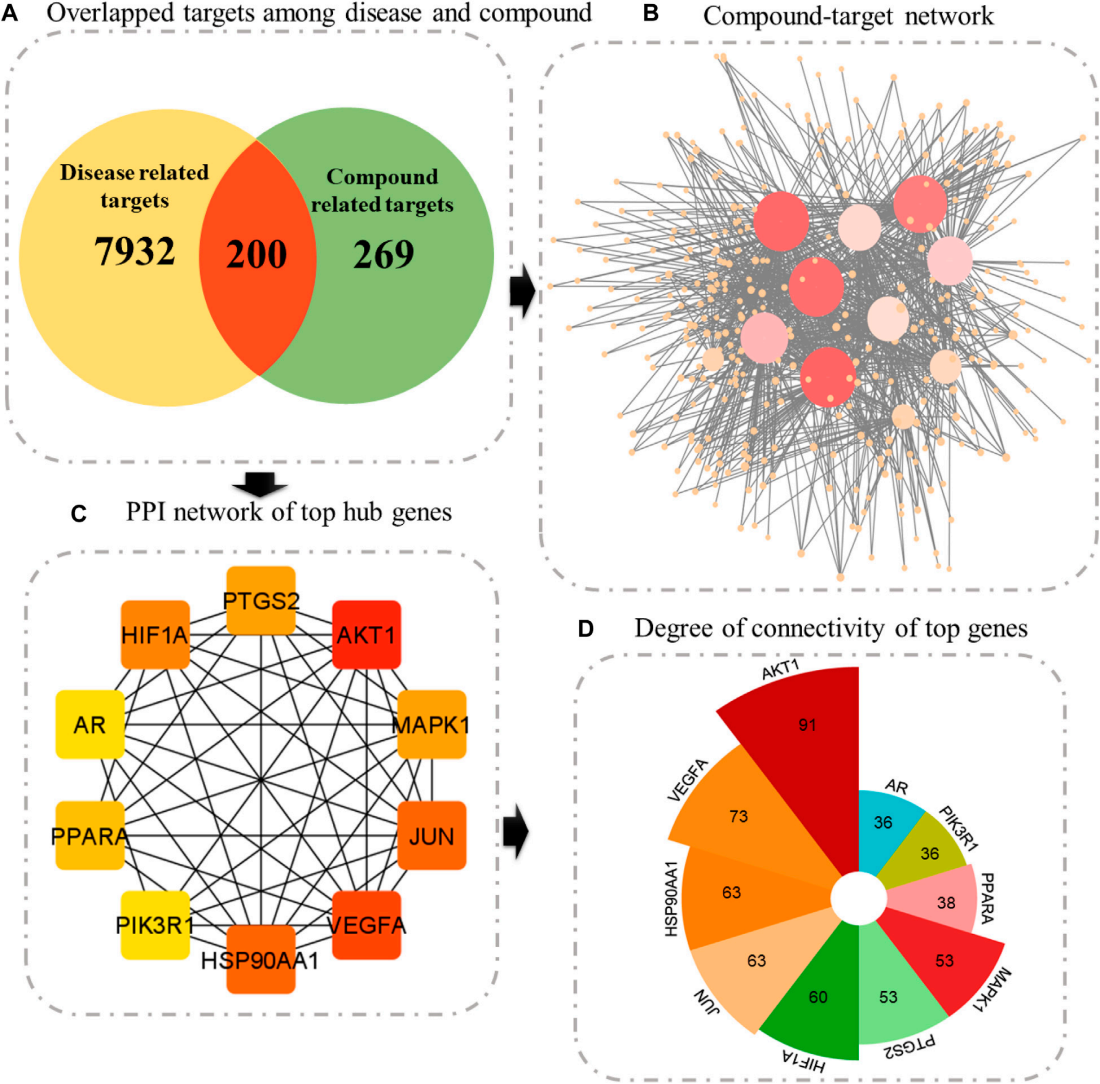
**(A)** Venn diagram illustrating overlapped targets, **(B)** compound–target network, where size signifies the degree of connectivity. **(C)** Top 10 genes ranked according to the degree algorithm. **(D)** Bar graph showing the degree of each central gene.

Constructing a PPI network is a fundamental step in network pharmacology, offering a structure to comprehend the intricate interactions between biological molecules and their impacts on disease pathways. Interactions among 200 common genes were predicted using the STRING database. This network comprised a total of 196 genes, forming 1,426 interactions with each other. One protein was found to interact with many proteins; therefore, the top 10 proteins with greater connectivity than other nodes were selected as hub genes. These genes are AKT1 (91), VEGFA (73), HSP90AA1 (63), JUN (63), HIF1A (60), PTGS2 (530, MAPK1 (53), PPARA (38), PIK3R1 (36), and AR (36) (Figure 4, Supplementary Material S6: Supplementary Table S1).

### 3.7 Compound–target protein–pathway network construction

**FIGURE 5:**
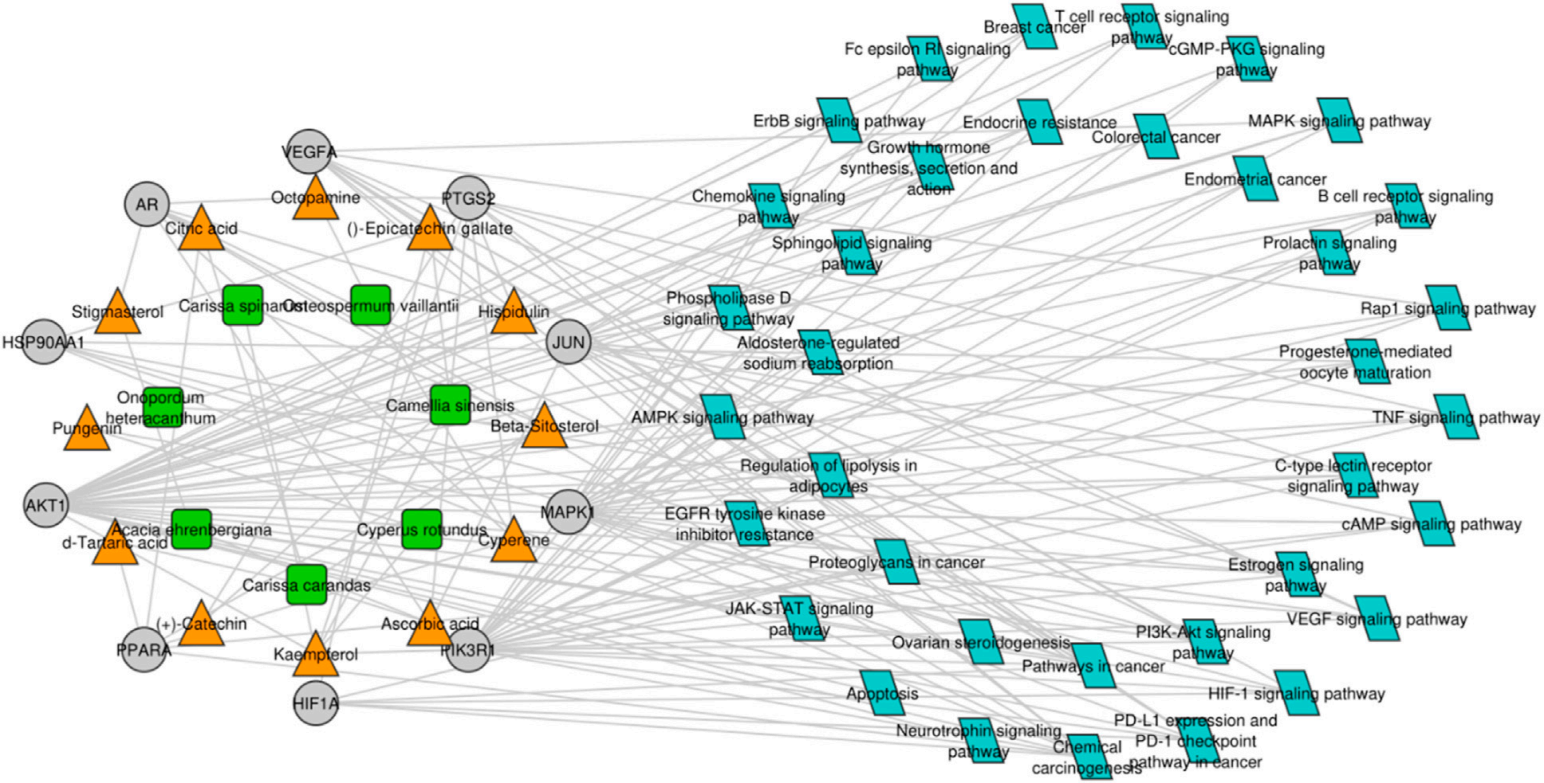
Compound–target protein–pathway network. The green and orange nodes represent the plants and their respective compounds, while the gray nodes represent the hub genes, and blue nodes indicate the pathways in which the hub genes are mainly involved.

For gaining a deep understanding of the underlying action mechanisms of indigenous plants in ovarian cancer, an integrated “active ingredient–target–pathway” network was constructed using the GO and KEGG pathway databases, and non-ovarian cancer-associated pathways were excluded from the analysis (Figure 5). Subsequently, a comprehensive analysis of the PPI network and active ingredient–target–pathway network was performed at the systematic level to identify top-ranked proteins, including AKT1 and VEGFA, which were further subjected to molecular docking analysis. AKT1 and VEGFA were selected as most of the compounds targeted these genes, and these genes are mainly linked to ovarian cancer-related pathways, including the estrogen signaling pathway, progesterone-mediated oocyte maturation, the prolactin signaling pathway, Rap1 and ErbB signaling pathways, colorectal cancer, growth hormone action, secretion, and synthesis, and endometrial cancer.

### 3.8 Molecular docking analysis

**FIGURE 6:**
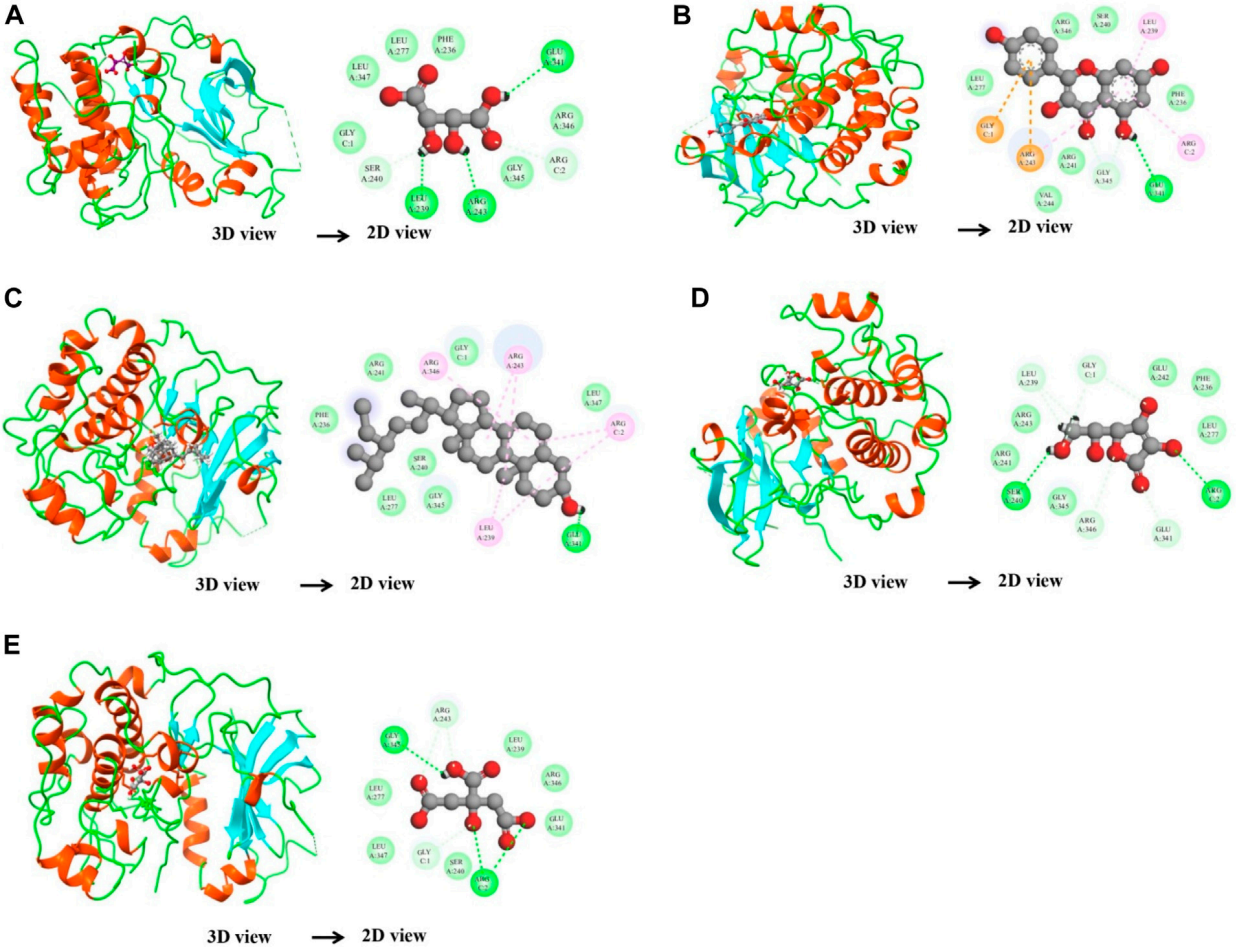
Binding interactions between the AKT1 protein and five different active compounds. Each panel **(A–E)** displays a 3D view of the protein–compound complex on the left and a corresponding 2D interaction diagram on the right. The compounds studied include ascorbic acid **(A)**, citric acid **(B)**, kaempferol **(C)**, stigmasterol **(D)**, and d-tartaric acid **(E)**. These diagrams highlight the specific amino acid residues of the AKT1 protein involved in the interaction with each compound, providing insights into the molecular docking and potential binding efficacy.

After network analysis, two proteins, AKT1 and VEGFA, were chosen for docking analysis, as these proteins were found to be targeted by multiple compounds and also linked to disease-relevant pathways. All selected compounds were docked against AKT1 and VEGFA, allowing for the assessment of their binding affinity, interaction stability, and free energy within the target protein’s active site. These proteins were docked with selected compounds; however, the top five compounds based on their binding affinity were selected. In the case of the AKT1 protein, the top five compounds with maximum binding affinities are ascorbic acid (−11.1227), citric acid (−10.2178), kaempferol (−11.4719), stigmasterol (−10.0388), and d-tartaric acid (−10.9147) (Figure 6). In the case of the AKT1–ascorbic acid complex, binding affinity contributed to H-bonds with the Ser A:240; Leu A:239; Arg A:243; Arg C:2; and Glu A:341 residues. In terms of citric acid, AKT1 formed H-bonds with Glu A:341 and Gly A:345; for kaempferol, AKT1 demonstrated H-bond interaction with Glu A:341; for stigmasterol, the H-bond interaction was found with Ser A:240 and Arg C:2; and d-tartaric acid showed H-bond interaction with Arg C:2; Glu A:341; Arg A:346; Gly C:1; and Leu A:239 residues (Table 4).

**TABLE 4:** Binding affinity and RMSD value of AKT1 with top five compounds.

Protein	Compound	Docking score (kcal/mol)	RMSD (Å)	Interacting residues
AKT1_3QKK	Ascorbic acid	−11.1227	0.804941	Ser:240; Leu:239; Arg:243; Arg:2; Glu:341
	Citric acid	−10.2178	1.313716	Glu:341; Gly:345; Gly:1; Arg:2; Arg:243; Leu:239
	Kaempferol	−11.4719	0.877178	Arg:346; Arg:243; Arg:2; Leu:239; Glu:341
	Stigmasterol	−10.0388	1.137621	Ser:240; Arg:346; Arg:341; Arg:2; Gly:1; Leu:239
	d-Tartaric acid	−10.9147	0.994647	Arg:2; Glu:341; Arg:346; Gly:1; Leu:239

**FIGURE 7:**
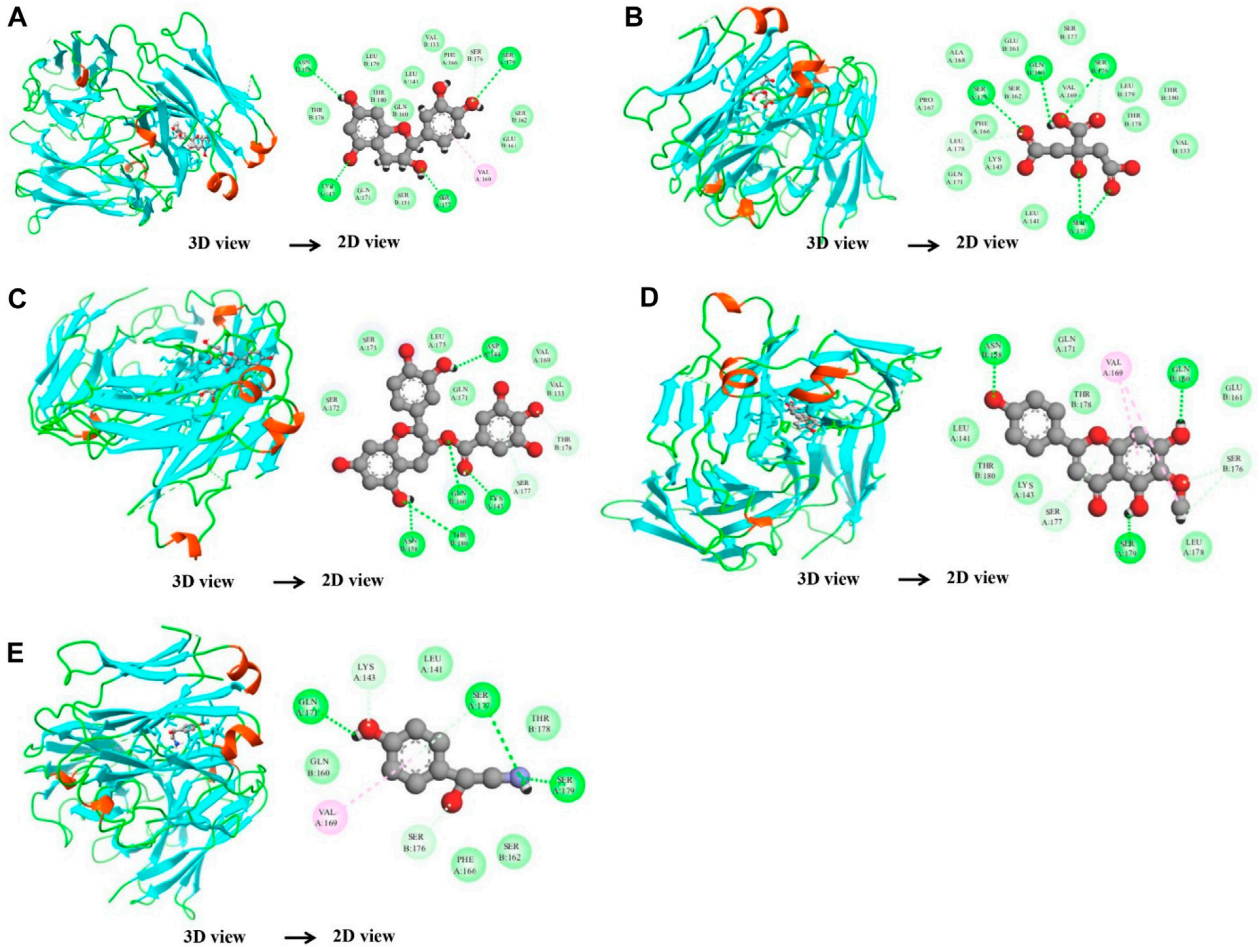
Molecular docking results of the VEGFA protein with five distinct compounds, showcasing the interaction details in both 3D and 2D representations. For each compound, the left side depicts the protein in a 3D structure with the bound compound, while the right side shows a 2D interaction map. The specific compounds analyzed are (+)-catechin **(A)**, citric acid **(B)**, (−)-epicatechin gallate **(C)**, hispidulin **(D)**, and octopamine **(E)**. The interaction maps highlight the key amino acids of the VEGFA protein that potentially contribute to the binding affinity and specificity for each compound.

In the case of the VEGFA protein, (+)-catechin (−15.6498), citric acid (−14.7792), (−)-epicatechin gallate (−15.1182), hispidulin (−17.3714), and octopamine (−17.517) were found to have more binding affinity than other active ingredients (Figure 7). In the case of the VEGFA-(+)–catechin complex, binding affinity contributed to H-bonds with Asn B:158; Ser A:179; Ser A:176; Lys A:143; and Ser A:177; for citric acid, VEGFA has H-bond interactions with Ser A:177; Ser A:179; Leu A:178; Gln B:160; Ser B:176 Ser A:177; Ser A:179; Leu A:178; Gln B:160; and Ser B:176; for (−)-epicatechin gallate, VEGFA demonstrated H-bond interactions with Asn A:158; Thr B:180; Gln B:160; Lys A:143; and Asp A:144; for hispidulin, H-bond interactions were found with Asn A:158; Gln B:160; Ser A:179; and Ser B:176; and octopamine showed H-bond interactions with Ser A:177; Ser A:179; Ser B:176; and Gln A:171 residues (Table 5).

**TABLE 5:** Binding affinity and RMSD value of VEGFA with top five compounds.

Protein	Compound	Docking score (kcal/mol)	RMSD (Å)	Interacting residues
VEGFA_4ZFF	(+)-Catechin	−15.6498	1.208914	Asn:158; Ser:179; Ser:176; Lys:143; Ser:177; Val:169
	Citric acid	−14.7792	1.159474	Ser:177; Ser:179; Leu:178; Gln:160; Ser:176
	(−)-Epicatechin gallate	−15.1182	1.816931	Asn:158; Thr:180; Gln:160; Lys:143; Asp:144
	Hispidulin	−17.3714	0.916578	Asn:158; Val:190; Gln:160; Ser:179; Ser:176
	Octopamine	−17.517	1.156475	Ser:177; Ser:179; Ser:176; Gln:171

### 3.9 MD simulation

**FIGURE 8:**
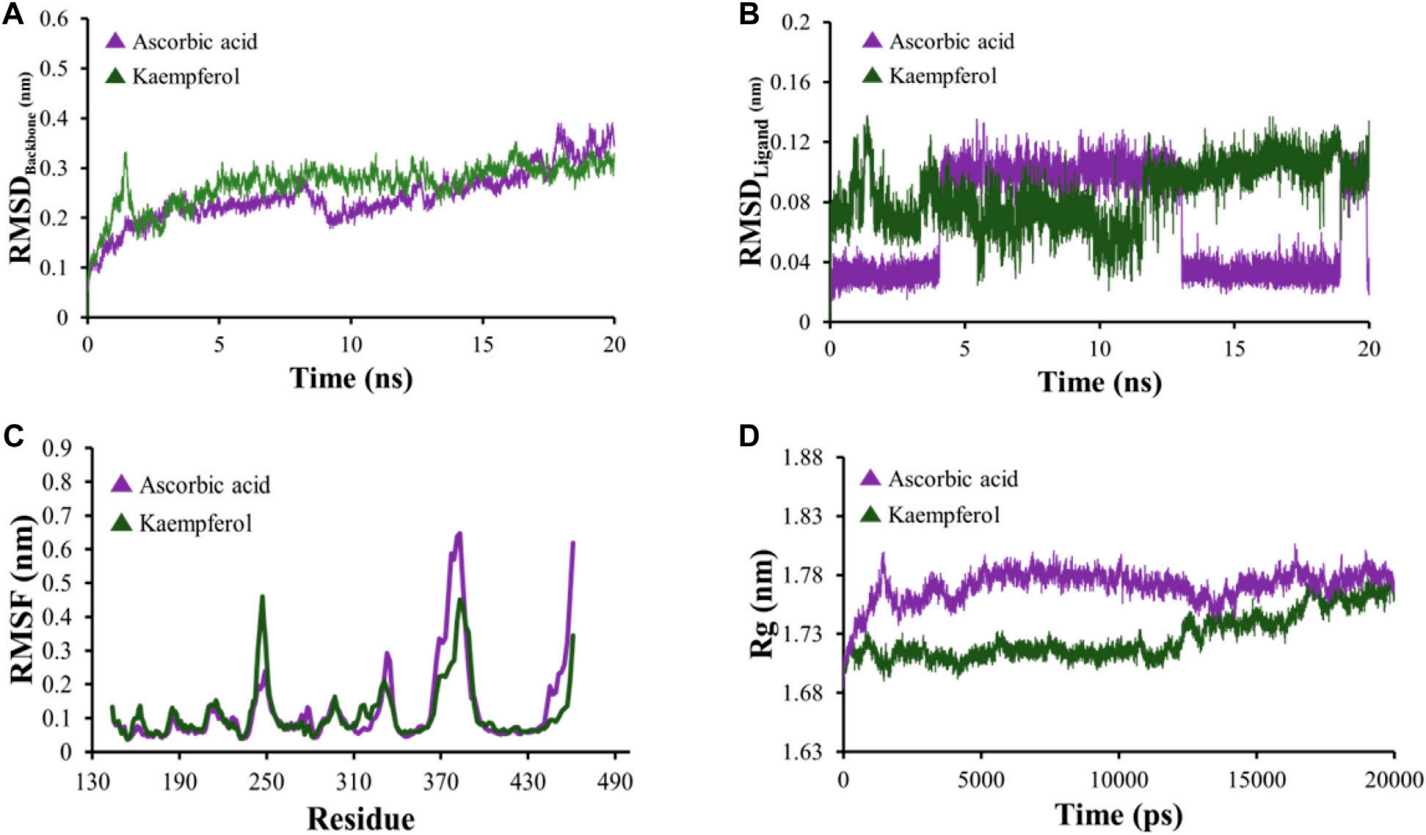
**(A)** RMSD analyses of backbone atoms (C, Cα, and N) within the AKT1–ligand complexes. **(B)** RMSD evaluations for ligand atoms in the AKT1–ligand systems. **(C)** RMSF measurements for backbone atoms in the AKT1–ligand setups. **(D)** Rg analysis pertaining to backbone atoms.

**FIGURE 9:**
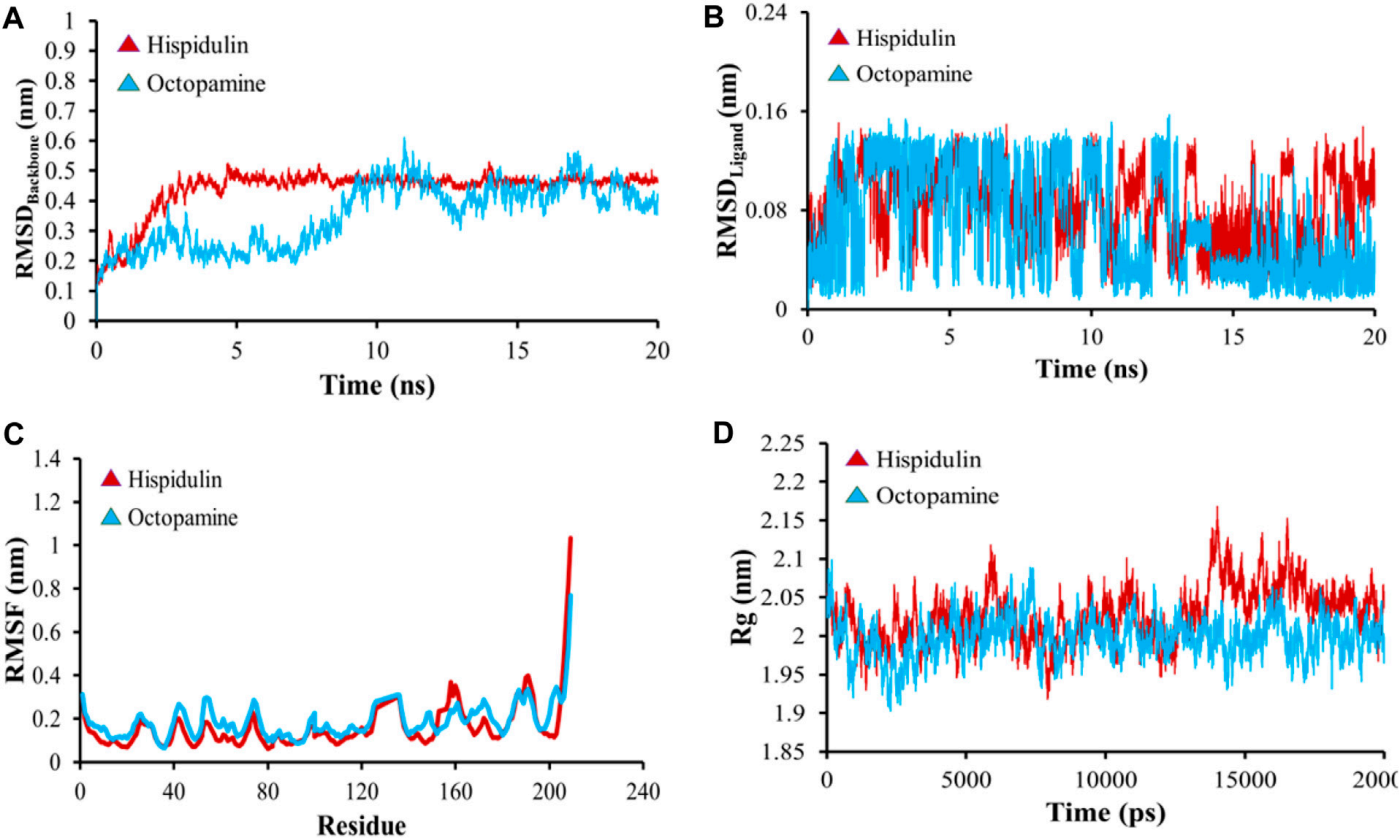
**(A)** RMSD analyses of backbone atoms (C, Cα, and N) within the VEGFA–ligand complexes. **(B)** RMSD evaluations for ligand atoms in the VEGFA–ligand systems. **(C)** RMSF measurements for backbone atoms in the VEGFA–ligand setups. **(D)** Rg analysis pertaining to backbone atoms.

All-atom Molecular Dynamics (MD) simulations were conducted to analyze the relationship between the AKT1 and VEGFA proteins and to assess the stability of the active compounds. The simulation was run at 20 ns using GROMACS software. For each protein, the top two docked complexes based on their binding energies were chosen for MD simulation for evaluating their RMSD and RMSF values, which assist in analyzing conformational changes and interactions among small molecules and target proteins. RMSD offers an understanding of the extent of deviation that a set of atoms (complexes, compounds, and proteins) experience from their original reference structure. On the other hand, RMSF calculates the temporal progression of the average discrepancy for each residue from its initial position in the minimized structures. The RMSD analysis revealed that selected complexes stabilized within a 20-ns timeframe. The average RMSD of the backbone atoms of AKT1 with kaempferol and ascorbic acid–protein systems was found to be ∼0.175 and ∼0.23Å, respectively (Figure 8A), while for VEGFA, the average RMSD for hispidulin and octopamine was found to be 0.45 and 0.5 Å, respectively (Figure 9A). On the other hand, the RMSD of ligand atoms for AKT1 with ascorbic acid exhibits an average RMSD value of ∼0.04, with a sharp increase in RMSD (∼0.12 Å) observed after 4–13 ns of simulation (Figure 8B), while for kaempferol, the RMSD falls within the range of ∼0.08 to ∼0.10 Å. In the case of the VEGFA protein, the average RMSD value for octopamine and hispidulin was ∼0.14 and ∼0.12 Å (Figure 9B). These findings suggest that octopamine was more stable at 20 ns in the case of the VEGFA protein, while for AKT1, kaempferol was found to be more stable. This was further substantiated by analyzing RMSF *versus* the residue number of AKT1, which showed that in comparison to AKT1–kaempferol, the AKT1–ascorbic acid complex exhibited greater fluctuations in backbone residues (Figure 8C). However, in terms of the VEGFA complex, hispidulin indicated greater fluctuations in backbone residues than octopamine (Figure 9C).

The radius of gyration (Rg) serves as a crucial metric to evaluate changes in the compactness of a docked complex. For AKT1, ascorbic acid exhibited more pronounced fluctuations than kaempferol (Figure 8D). In the context of VEGFA, notably, the Rg values for VEGFA–hispidulin exhibited a narrower distribution, denoting a more consistent structural compactness relative to the VEGFA–octopamine complex, which displayed a broader range of Rg values, suggestive of increased flexibility and structural variability. This differential behavior in Rg implies that the binding of hispidulin may confer a greater degree of conformational restraint upon the VEGFA protein, potentially influencing the stability and functional specificity of the ligand–protein interaction (Figure 9D). Furthermore, insights derived from the evaluation of Rg, RMSF, and RMSD metrics indicate that all complexes maintained consistent stability throughout the simulation period. Therefore, it could be concluded that these small molecules hold the potential to be used as an inhibitor for AKT1 and VEGFA: however, the finding needs further *in vivo* and *in vitro* validation.

## 4 Discussion

According to the National Cancer Registry in Saudi Arabia, ovarian cancer is the seventh most commonly diagnosed cancer among women, accounting for 3.3% of all cancers (Younes and Zayed, 2019). In 2020, the age-standardized incidence rate and mortality rate of ovarian cancer in Saudi Arabia were 3.8/100,000 women and 2.7/100,000 women, respectively. Additionally, 444 new cases were reported in 2020, constituting 1.6% of new cancer cases in Saudi Arabia, with 281 of those cases resulting in death (Aga et al., 2022). Unfortunately, effective treatment options for ovarian cancer are limited in the country. As a result, there is a critical need for the development of effective treatment options to fight against ovarian cancer. Research efforts aimed at identifying new therapeutic targets as well as improving existing treatments are highly recommended.

The utilization of natural products for the treatment of cancer is widely recognized and has garnered significant attention from researchers worldwide (Pangestuti and Kim, 2011). Saudi Arabia has a rich flora consisting of numerous plant species, some of which have been traditionally used in natural medicine (Aati et al., 2019). Many of these plants possess bioactive compounds that have shown potential in cancer treatment. For example, *C. sinensis* is a rich source of polyphenols associated with numerous pharmacological properties such as anti-inflammatory, antimicrobial, and anti-aging (Rahmani et al., 2015; Naveed et al., 2018). With continued research, these and other Saudi Arabian plants may prove to be valuable sources of natural compounds for the development of new cancer therapies. Recent studies reported that local plant extracts from Saudi Arabia, including *O. heteracanthum* (Gouda et al., 2014), *A. ehrenbergiana* (Makeen et al., 2020), *O. vaillantii* (Gouda et al., 2014), *C. rotundus* (AlQathama et al., 2022), *C. carandas* (Alshehri, 2020), *C. spinarum* (AlQathama et al., 2022), and *C. sinensis* (Dou et al., 2021), exhibited remarkable and potent cytotoxic effects on the ovary cancer cell line. Nevertheless, the specific mechanisms of action underlying these medicinal plants remain unclear. The current study focused on identifying potential active compounds of these Saudi Arabian local plants as a new effective treatment option against ovarian cancer. Initially, the information related to the phytochemicals of *O. heteracanthum*, *A. ehrenbergiana*, *O. vaillantii*, *C. rotundus*, *C. carandas*, *C. spinarum*, and *C. sinensis* was retrieved from databases and reported in a literature survey. Later, the microarray data from the GSE54388, GSE69428, GSE36668, and GSE40595 datasets were obtained from GEO databases and screened for the identification of DEGs. The preprocessing of microarray data was performed through the limma package of R for the identification of DEGs. Later, the disease-related data were compared with plant-related data, which demonstrated 200 overlapped targets. These common targets were then considered for further network pharmacology. The functional annotation of these putative targets revealed that these genes are mainly involved in growth hormone action, secretion, and synthesis, pathways in cancer, the estrogen signaling pathway, pI3K-Akt and ErbB signaling pathways, colorectal cancer, breast cancer, the Rap1 signaling pathway, progesterone-mediated oocyte maturation, endometrial cancer, and the prolactin signaling pathway.

In ovarian cancer, estrogen signaling can promote tumor growth and progression through several mechanisms. For instance, estrogen can promote the growth of ovarian cancer cells and enhance their resistance to chemotherapy (Choi et al., 2011). Additionally, estrogen signaling can promote the formation of new blood vessels, which helps supply the tumor with nutrients and oxygen (Ribeiro and Freiman, 2014). Furthermore, estrogen signaling can interact with other pathways that are implicated in ovarian cancer, such as the PI3K/AKT/mTOR pathway (Langdon et al., 2020). This pathway assists in regulating cell growth, survival, and metabolism and is often dysregulated in ovarian cancer. Estrogen signaling can activate the PI3K/AKT/mTOR pathway, leading to further promotion of tumor growth and survival (Gil, 2014). Therefore, by directing efforts toward the genes active in PI3K-Akt pathways, the progression of ovarian cancer can be halted.

After functional annotation, AKT1 and VEGFA emerged as pivotal proteins due to their highest connectivity within the PPI network, compound–target network, and compound–target–disease network. Although molecular docking provides an estimation of compound suitability within the protein’s active site, it solely provides information at the protein’s active site. Hence, to analyze the compound–protein target system, the use of binding conformation data has become more prevalent, necessitating the application of MD simulations and their associated binding energy measurements. MD simulations facilitate in-depth exploration of the dynamic attributes of docked complexes as well as fluctuations in the energy scenario. These insights are crucial in determining the complex’s stability and possible structural shifts in the protein initiated by ligand interaction. Overall, the assessment derived from molecular docking and MD simulations underscored a considerable binding affinity in the interactions involving active compounds and proteins.

Previous studies reported that AKT1 and VEGFA have key roles to play in the pathogenesis of ovarian cancer. AKT1 is a serine/threonine kinase that is frequently activated in ovarian cancer, and it is involved in promoting cell survival, proliferation, and migration (Tokunaga et al., 2008). When activated, AKT1 can stimulate the expression of VEGFA, which is a key pro-angiogenic factor that promotes the formation of new blood vessels (Hsieh et al., 2017; Zhu et al., 2018). In ovarian cancer, the upregulation of AKT1 and VEGFA is associated with proliferation, metastasis, and angiogenesis (Wang et al., 2019). Moreover, AKT1 and VEGFA can interact with each other, forming a positive feedback loop that further promotes ovarian cancer progression (Trinh et al., 2009). AKT1 activates the HIF-1α regulator, which in turn can upregulate VEGFA expression (Mazure et al., 2003). VEGFA, in turn, activates the AKT1 pathway, creating a self-reinforcing loop that promotes tumor growth and angiogenesis. Thus, targeting the AKT1 and VEGFA signaling pathways is a promising approach for treating ovarian cancer. Inhibition of AKT1 and VEGFA signaling has been shown to reduce ovarian cancer cell proliferation, angiogenesis, and tumor growth in preclinical models. Furthermore, several clinical trials are currently needed to investigate the efficacy of AKT1 and VEGFA inhibitors in the treatment of ovarian cancer.

Recent advancements in the study of phytochemicals and their role in cancer therapy have underscored the potential of natural compounds as effective agents against ovarian cancer. For instance, Lu et al. (2023) highlighted the emerging role of plant-derived compounds in modulating key signaling pathways involved in cancer progression, including the PI3K-Akt and VEGFA pathways, which our research also identifies as critical targets. Furthermore, Song et al. (2015) reported the significant *in vivo* efficacy of kaempferol in reducing tumor growth and metastasis through the inhibition of cancer-related signaling pathways. These findings align with our computational predictions and molecular docking results, suggesting a promising avenue for future research to explore these compounds’ clinical applications. Moreover, the integration of bioinformatics tools and network pharmacology in recent studies, as demonstrated by Batool et al. (Alshehri, 2020), has facilitated a deeper understanding of the complex interactions between natural compounds and cancer-related pathways, reinforcing the value of our approach in identifying novel therapeutic candidates. These contemporary findings not only validate our methodology but also indicate a growing consensus on the importance of targeting specific molecular pathways in ovarian cancer treatment, providing a robust framework for future experimental and clinical investigations.

To sum up, our investigation lays the groundwork for uncovering the multi-target effect of native Saudi Arabian plants as potential therapeutic agents for ovarian cancer. The combination of network pharmacology and bioinformatics methodologies aids in pinpointing essential interactions and molecular pathways contributing to ovarian cancer and supports the identification of prospective drug targets to combat this disease. Although we corroborated our findings using molecular docking and MD simulations, additional *in vitro* and *in vivo* studies are required to substantiate the efficacy of our results. Our study bears certain limitations, such as the requirement for further experimental validation of our findings, the need for a more comprehensive database of traditional medicinal plants and target genes to enhance the precision of network pharmacology analysis results, and the incomplete understanding of the exact therapeutic mechanism employed by local plants in the treatment of ovarian cancer, even after merging the results from network pharmacology and molecular docking. Therefore, multidisciplinary integration is essential to fully grasp the operational mechanism of these local plants in relation to ovarian cancer.

## 5 Conclusion

The significant prevalence of ovarian cancer in Saudi Arabia underscores the urgent need for effective prevention, early detection, management strategies, and addressing its associated complications. In response, our study presents an innovative scientific framework that explores the complex multi-target interactions of active phytochemicals within native Saudi Arabian plants. By integrating bioinformatics with network pharmacology approaches, we have identified several potential compounds, including hispidulin, stigmasterol, ascorbic acid, octopamine, cyperene, kaempferol, pungenin, citric acid, d-tartaric acid, beta-sitosterol, (−)-epicatechin gallate, and (+)-catechin, that show promise in treating ovarian cancer. Our findings also highlight the therapeutic implications of targeting AKT1 and VEGFA pathways to mitigate cellular proliferation and growth, contributing valuable insights into the chemical constituents of indigenous Saudi plants and their synergistic mechanisms against ovarian cancer. Looking forward, our research paves the way for further in-depth studies to validate the efficacy and safety of these identified phytochemicals through clinical trials. Future work will also explore the optimization of these compounds for better bioavailability and specificity, along with investigating combination therapies to enhance their anti-cancer effects. Additionally, the development of more sophisticated bioinformatics tools and network pharmacology models will be crucial in unraveling the complexities of ovarian cancer pathogenesis and the multi-faceted roles of these compounds. Ultimately, our study lays the groundwork for novel therapeutic strategies that could significantly impact the prevention and treatment of ovarian cancer in Saudi Arabia and beyond.

## Data Availability

The original contributions presented in the study are included in the article/Supplementary Material; further inquiries can be directed to the corresponding author.
