# Supplementary material for: From desert flora to cancer therapy: systematic exploration of multi-pathway mechanisms using network pharmacology and molecular modeling approaches
Source: Front Pharmacol. 2024 Apr 11;15:1345415. doi: 10.3389/fphar.2024.1345415 (PMC11043532; doi:10.3389/fphar.2024.1345415)
Supplement: Supplementary file 1 [file DataSheet1.ZIP › Supplementary File 7.docx]

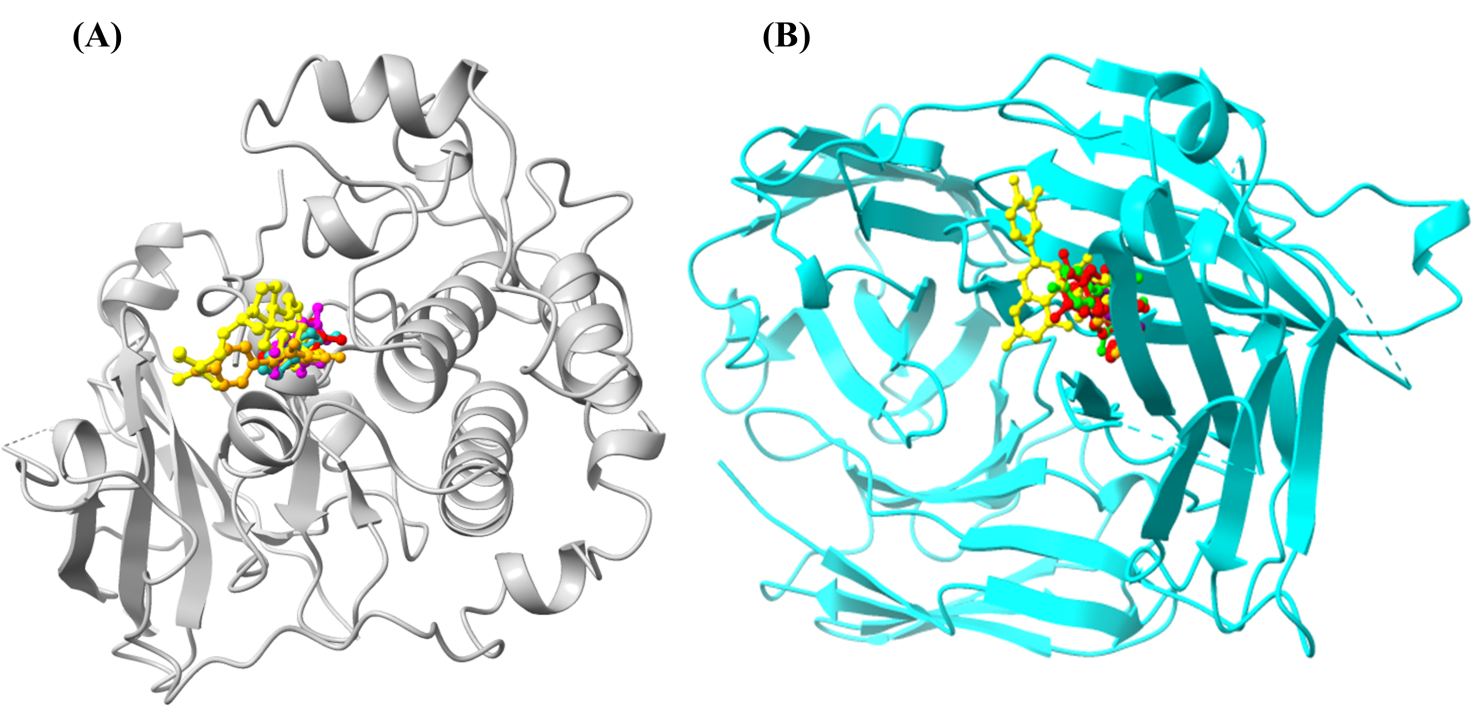


**Figure S1:** Visual representation of ligand-protein complexes: Panel (A) depicts the AKT1 protein in a complex with a ligand, illustrated in a ribbon diagram with the ligand rendered in ball-and-stick model highlighted in yellow and magenta. Panel (B) shows the VEGFA protein bound to a distinct ligand, also presented in a ribbon diagram with the ligand in ball-and-stick format, but with atoms color-coded to emphasize different elements. Both structures exemplify the three-dimensional conformation and potential binding pockets of the respective proteins targeted by small molecule inhibitors.
